# Associations of Maternal Dietary Patterns during Pregnancy with Offspring Adiposity from Birth Until 54 Months of Age

**DOI:** 10.3390/nu9010002

**Published:** 2016-12-22

**Authors:** Ling-Wei Chen, Izzuddin M. Aris, Jonathan Y. Bernard, Mya-Thway Tint, Airu Chia, Marjorelee Colega, Peter D. Gluckman, Lynette Pei-Chi Shek, Seang-Mei Saw, Yap-Seng Chong, Fabian Yap, Keith M. Godfrey, Rob M. van Dam, Mary Foong-Fong Chong, Yung Seng Lee

**Affiliations:** 1 Department of Paediatrics, Yong Loo Lin School of Medicine, National University of Singapore, Singapore 119228, Singapore; paeclw@nus.edu.sg (L.-W.C.); lynette_shek@nuhs.edu.sg (L.P.-C.S.); 2 Singapore Institute for Clinical Sciences, Agency for Science, Technology and Research, Singapore 117609, Singapore; Izzuddin_Aris@sics.a-star.edu.sg (I.M.A.); Jonathan_Bernard@sics.a-star.edu.sg (J.Y.B.); marjorelee_colega@sics.a-star.edu.sg (M.C.); pd.gluckman@auckland.ac.nz (P.D.G.); yap_seng_chong@nuhs.edu.sg (Y.-S.C.); 3 Department of Obstetrics & Gynaecology, Yong Loo Lin School of Medicine, National University of Singapore, Singapore 119228, Singapore; obgmtt@nus.edu.sg (M.-T.T.); chiaairu@u.nus.edu (A.C.); 4 Liggins Institute, University of Auckland, Auckland 1023, New Zealand; 5 Saw Swee Hock School of Public Health, National University of Singapore, Singapore 117549, Singapore; seang_mei_saw@nuhs.edu.sg (S.-M.S.); rob_martinus_van_dam@nuhs.edu.sg (R.M.v.D.); 6 Department of Pediatric Endocrinology, KK Women’s and Children’s Hospital, Singapore 229899, Singapore; fabian.yap.k.p@singhealth.com.sg; 7 Duke-NUS Graduate Medical School, Lee Kong Chian School of Medicine, Singapore 169857, Singapore; 8 MRC Lifecourse Epidemiology Unit & NIHR Southampton Biomedical Research Centre, University of Southampton & University Hospital Southampton NHS Foundation Trust, Southampton SO16 6YD, UK; kmg@mrc.soton.ac.uk; 9 Department of Medicine, Yong Loo Lin School of Medicine, National University of Singapore, Singapore 119228, Singapore; 10 Department of Nutrition, Harvard School of Public Health, Boston, MA 02115, USA; 11 Clinical Nutrition Research Centre, Singapore Institute for Clinical Sciences, A*STAR, Singapore 117599, Singapore; 12 Khoo Teck Puat-National University Children’s Medical Institute, National University Health System, Singapore 119228, Singapore

**Keywords:** pregnancy, dietary patterns, developmental origins of health and diseases, children, adiposity, BMI, subscapular skinfold, triceps skinfold, fruit, vegetables

## Abstract

Most studies linking maternal diet with offspring adiposity have focused on single nutrients or foods, but a dietary pattern approach is more representative of the overall diet. We thus aimed to investigate the relations between maternal dietary patterns and offspring adiposity in a multi-ethnic Asian mother–offspring cohort in Singapore. We derived maternal dietary patterns using maternal dietary intake information at 26–28 weeks of gestation, of which associations with offspring body mass index (BMI), abdominal circumference (AC), subscapular skinfold (SS), and triceps skinfold (TS) were assessed using longitudinal data analysis (linear mixed effects (LME)) and multiple linear regression at ages 0, 3, 6, 9, 12, 15, 18, 24, 36, 48, and 54 months. Three dietary patterns were derived: (1) vegetables-fruit-and-white rice (VFR); (2) seafood-and-noodles (SfN); and (3) pasta-cheese-and-bread (PCB). In the LME model adjusting for potential confounders, each standard deviation (SD) increase in maternal VFR pattern score was associated with 0.09 mm lower offspring TS. Individual time-point analysis additionally revealed that higher VFR score was generally associated with lower postnatal offspring BMI *z*-score, TS, SS, and sum of skinfolds (SS + TS) at ages 18 months and older. Maternal adherence to a dietary pattern characterized by higher intakes of fruit and vegetables and lower intakes of fast food was associated with lower offspring adiposity.

## 1. Introduction

The prevalence of childhood obesity has risen at an alarming rate [1]. Overweight and obese children are not only more likely to become overweight and obese as adults, but also have higher risk of stroke, heart disease, and type 2 diabetes in adulthood [2]. In the recent Commission of Ending Childhood Obesity report, it was highlighted that as of 2014, approximately 41 million children under five years old are either obese or overweight, with close to half of them living in Asia [3,4]. In addition to the promotion of healthy food intakes and physical activity during childhood, the importance of prenatal care, including appropriate maternal nutrition, was identified as a key strategy in the prevention of childhood obesity [4].

The theory of in utero exposure having a lifelong influence on offspring health, initially proposed by Barker and colleagues [5], has been increasingly substantiated by evidence from both epidemiological and experimental studies [6]. For example, it has been reported that in utero famine exposure is associated with increased risks of obesity [7], coronary heart disease [8], and hypertension [9] during adulthood. More recent studies also suggested that less severe prenatal nutritional challenges (e.g., suboptimal macronutrient balance [10]) can impart long-term influence on offspring, with epigenetic alterations proposed to be a major underlying mechanism [11].

To date, most nutritional epidemiological studies investigating associations between maternal nutrition and offspring birth outcomes or body composition have used single nutrient and food approaches. While useful in isolating influences of specific foods or nutrients, these approaches may not be adequate to account for the complex behavior of food consumption and interactions among nutrients [12]. The dietary pattern approach may be easier to interpret by the public and thus more useful for public health messaging [12]. In two large cohort studies (*n* > 40,000), maternal dietary patterns characterized by high intakes of meats, fats, and potatoes were associated with increased risks of adverse birth outcomes, while patterns characterized by high intakes of vegetables, fruit, fish, and poultry were associated with a decreased risk [13,14]. Of two existing studies examining the associations with offspring postnatal body composition, one found that higher maternal adherence to “processed foods” pattern was associated with higher risk of offspring overweight and obesity at five years of age [15], while the other showed no association [16].

The relationship between maternal dietary patterns and offspring body composition remains unclear and it remains to be elucidated whether having longitudinal offspring postnatal measurements at multiple time-points can reveal relationships that are otherwise not apparent in single time-point analyses. Thus far, the relationships between maternal dietary patterns and offspring adiposity have not been examined in Asian populations, where dietary patterns can be vastly different due to social and cultural differences [12] and where the risk of metabolic disorders such as type 2 diabetes is higher than in Caucasian populations at similar BMI levels [17,18,19]. We aimed to investigate this by using longitudinal analysis of repeated offspring anthropometric measurement data derived from the Growing Up in Singapore Towards healthy Outcomes (GUSTO) study, a multi-ethnic Asian mother–offspring cohort.

## 2. Materials and Methods

### 2.1. Study Design

GUSTO is a mother–offspring cohort study that has been described in detail elsewhere [20]. Briefly, from June 2009 to September 2010, pregnant women attending antenatal care visits (<14 weeks of gestation) were recruited from the KK Women’s and Children’s Hospital (KKH) and National University Hospital (NUH), the major public maternity units in Singapore. To be eligible, the pregnant women had to be Singapore citizens or permanent residents of homogeneous ethnic background (Chinese, Malay, or Indian) and between 18 and 50 years old, to agree to donate cord blood, cord, and placenta, and intending to deliver in KKH and NUH and to reside in Singapore for the next 5 years. Women with serious health conditions such as psychosis and type 1 diabetes mellitus were excluded. The study was granted ethical approval by both the National Healthcare Group Domain Specific Review Board (reference No. D/09/021) and the Sing Health Centralized Institutional Review Board (reference No. 2009/280/D). Written informed consent was collected from all women at recruitment.

### 2.2. Subjects

**Figure 1:**
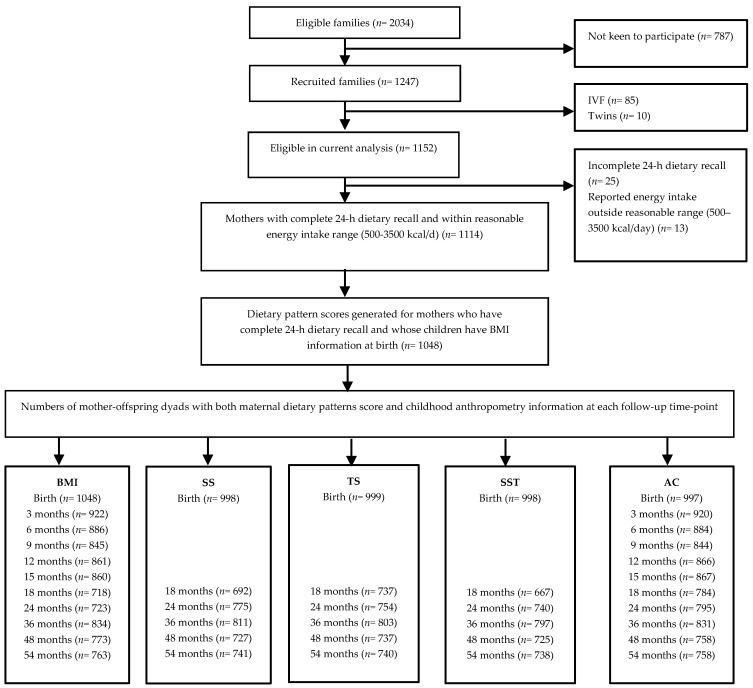
Participant flow chart. BMI, body mass index; SS, subscapular skinfold; TS, triceps skinfold; SST, sum of skinfolds thickness; AC, abdominal circumference.

Of 2034 eligible women, 1247 consented and were recruited into the study. Pregnant women who underwent in vitro fertilization or were bearing twins (*n* = 95) were excluded from the present analysis. From the remaining 1152, 1127 completed a single 24-h dietary recall and 1087 babies were delivered. We further excluded 13 mothers with unrealistic reported energy intake (<500 kcal/day or >3500 kcal/day) [21]. Of the remaining mother–child dyads, 1048 had information on both maternal dietary intake and offspring BMI at birth. The extraction of maternal dietary patterns and baseline information was thus limited to this subset. The participant flow is detailed in Figure 1. Numbers of mother–offspring dyads with both maternal dietary pattern and childhood anthropometry information at each follow-up time-point were 718–1048 for body mass index (BMI), 692–998 for subscapular skinfold (SS), 737–999 for triceps skinfold (TS), and 758–997 for abdominal circumference (AC).

### 2.3. Maternal Dietary Assessment and Extraction of Dietary Patterns

Assessment of maternal dietary intakes in this cohort has been described in detail elsewhere [22,23]. Briefly, clinical research staff conducted 24-h dietary recalls at 26–28 weeks gestation with a 5-step, multiple-pass interviewing method [24] using visual aids to assist women in quantifying their dietary intakes. Nutrient analysis of the dietary recalls was performed using nutrient analysis software (Dietplan6, Forestfield Software, West Sussex, UK) and a food composition database of locally available foods [25], with modifications made to inaccuracies found. All food and drinks recorded were subsequently grouped according to similarity of nutrient composition and comparable usage (e.g., apple, orange, watermelon, etc. were grouped into the “fruit” food group), resulting in 68 food groups (Supplementary Materials Table S1). Dietary patterns were derived by exploratory factor analysis with varimax rotation on the 68 food groups. Exploratory factor analysis is an established technique to extract dietary patterns [26,27]; it reduces the dimension of the data by aggregating correlated variables, resulting in linear combinations (factors) of the included variables [26,27,28]. Three factors (i.e., dietary patterns) were retained based on break point of the Scree plot (Supplementary Materials Figure S1), eigenvalues >1.5, and factor interpretability. Dietary pattern scores for each participant were then calculated by summing the standardized intakes of food groups (g/day) weighted by their factor loadings (correlation coefficients between each food group and the dietary pattern).

Table 1 shows the factor loadings of specific food groups for the maternal dietary patterns. The top four positive loadings unique to the dietary pattern were used to label the pattern. The vegetables-fruit-and-white rice (VFR) pattern was characterized by high consumption of fruit, vegetables, and white rice and low consumption of fast food items and flavored rice; this pattern is reminiscent of a prudent pattern identified in many studies [29,30,31], including in a Singapore middle-aged population [32]. The seafood-and-noodles (SfN) pattern was characterized by high intakes of noodles, seafood, and soya sauce based gravies and low intakes of curry and ethnic bread; this pattern is reflective of the typical Chinese diet. Finally, the pasta-cheese-and-bread (PCB) pattern was defined by high consumption of pasta, cheese, bread, and butter, reflecting high intakes of Western food items.

In a small subset of participants (*n* = 212), information about late pregnancy maternal diet using a 3-day food diary was also available; we have previously shown that similar dietary patterns were extracted from this subset, and that the correlation coefficients of the dietary pattern scores were moderately strong (Pearson’s correlation coefficients (*r*) > 0.5, *p* < 0.001) [33,34]. To keep consistent with the analysis methods used in the previous publications [33,34], and to increase statistical power, we thus presented results for 24-h recall in this manuscript.

### 2.4. Maternal Characteristic

Data on maternal age, ethnicity, education level, and self-reported pre-pregnancy weight were collected from the participants at recruitment. During a clinic visit at 26–28 weeks of gestation, maternal weight (SECA weighing scale model 803, SECA Corp., Hamburg, Germany) and height (SECA stadiometer model 213) were measured, and weight gain up to 26–28 weeks of gestation was derived by subtracting self-reported pre-pregnancy weight from the weight at 26–28 weeks. Pre-pregnancy BMI was calculated as reported pre-pregnancy weight divided by the mother’s measured height squared (kg/m^2^). At the same clinic visit, maternal cigarette smoking, alcohol drinking, and physical activities were assessed using a questionnaire while maternal blood was collected for analysis of plasma vitamin D and folate concentrations. Furthermore, oral glucose tolerance tests were administered and gestational diabetes mellitus (GDM) was defined based on the 1999 World Health Organization criteria [35,36].

### 2.5. Child Characteristics

Information on birth weight, gestational age, infant sex, and birth order was abstracted from obstetric records. Gestational age was determined based on a dating ultrasound scan in the first trimester. Infant milk feeding was ascertained using interviewer-administered questionnaires at ages 3, 6, 9, and 12 months, and duration of any breastfeeding was subsequently calculated. Infant dietary intake (from which macronutrient and energy intakes were derived) was assessed at age 1 year using either a 24-h recall or a food diary. At age 2 years, the times children spend doing outdoor activities (e.g., walking and bike riding) and using media (e.g., watching television and playing video games) were assessed using a parental-report questionnaire.

Anthropometric measurements of offspring (weight, length/height, and AC) were obtained at birth and at ages 3, 6, 9, 12, 15, 18, 24, 36, 48, and 54 months. Weight until 18 months of age was measured using calibrated mobile digital baby scale (SECA model 334) to the nearest 1 g. After age 18 months, offspring weight was measured using calibrated digital scale (SECA model 813) to the nearest 10 g. From birth to age 24 months, recumbent length of the infants was measured from the top of the head to the soles of the feet using a mobile infant mat (SECA model 210) to the nearest 0.1 cm. From ages 18 months to 54 months, offspring head-to-heel standing height was measured using a stadiometer (SECA model 213). When both offspring length and height were present, the measurements were averaged. AC was measured using an inelastic measuring tape (Butterfly brand, China) and recorded to the nearest 1 mm. All anthropometric measurements were taken in duplicates (and subsequently averaged) using standardized protocols [37].

TS and SS were measured using Holtain skinfold calipers (Holtain Ltd., Crymych, UK) on the right side of the body and recorded to the nearest 0.2 mm at birth and at ages 18, 24, 36, 48, and 54 months; three measurements were taken with the two closest values averaged. Sum of skinfolds thickness (SST) were derived by adding TS and SS.

Anthropometric training and standardization sessions were conducted every 3 months, and observers were trained to obtain measurements that, on average, were the closest as possible to the values measured by an expert anthropometrist. Reliability was estimated by inter-observer technical error of measurement and coefficient of variation (Supplementary Materials Table S2). Offspring BMI was calculated using the formula weight (kg)/length^2^ (m^2^) and was subsequently transformed into age- and sex-specific *z*-score using a local Singapore reference [38].

### 2.6. Statistical Analysis

The dietary pattern score was used as continuous variable or in quartiles; a higher score or quartile indicates greater adherence to the dietary pattern.

Maternal and child characteristics were first summarized (mean ± SD or *n* (%)) according to quartiles of maternal dietary pattern scores. *p*-trends for the associations between maternal dietary pattern scores and maternal and child characteristics were assessed by modeling the median values of the quartiles in linear regression analysis for continuous variables and by Cochran–Mantel–Haenszel tests for categorical variables.

The longitudinal associations of maternal dietary pattern scores with offspring adiposity from birth through 54 months were examined using linear mixed effects (LME) models with an unstructured covariance matrix for random effects variables (intercept and slope) and maximum likelihood estimation method. The LME model takes into account within-subject correlation of repeated measurements and at the same time allows for incomplete outcome measurement [39]. Model selection was guided by Akaike and Bayesian information criteria, and the final LME model included linear, quadratic, and cubic terms for children’s age to estimate the change in adiposity indicators over time associated with a SD increase in dietary pattern scores. In addition to the fixed effect of age, we also allowed for a random intercept and random linear slope for age. Previous studies have suggested that the relationship between maternal diet and offspring adiposity may be modified by offspring sex and ethnicity [23,40,41]. Therefore, we tested interactions of maternal dietary pattern scores with offspring sex and ethnicity in relation to associations with measures of offspring adiposity by including the corresponding interaction terms into the models. Furthermore, we also assessed the maternal dietary pattern scores vs. offspring adiposity relationship at each time-point (at birth and at ages 3, 6, 9, 12, 15, 18, 24, 36, 48, and 54 months) using traditional linear regression as a complementary analysis. The analysis was first minimally adjusted for child’s age at the time of anthropometric measurement. The full model was further adjusted for potential confounders or determinants of childhood adiposity: (continuously) maternal age, height, pre-pregnancy BMI, weight gain until 26–28 weeks gestation, energy intake and scores of the other two dietary patterns, and infant gestational age at birth and duration of any breastfeeding; (categorically) maternal education level, ethnicity, gestational diabetes, and infant sex and birth order. Offspring sex and birth order are included in the model because they have been shown to affect childhood adiposity and obesity risk (thus, they are covariates in our analysis) [42,43,44].

We conducted several sensitivity analyses. First, we further adjusted our LME models for maternal smoking, alcohol intake, physical activities, and plasma vitamin D and folate concentrations. Second, to determine if the influence of maternal dietary patterns was independent of postnatal environment, the analyses were further adjusted for infant dietary intakes (macronutrient intakes) at age 1 year and duration of outdoor activities and media use at age 2 years. Third, we further included small-for-gestational age (birth weight <10th percentile for gestational age, calculated using a global reference for birth weight percentiles [45]), low birth weight (birth weight <2500 g), or total gestational weight gain (measured weight at last antenatal visit (mean ± SD gestational age: 37.3 ± 2.5 weeks) minus pre-pregnancy weight) as covariates in our models to assess potential mediation effects. Fourth, we excluded participants with preeclampsia (*n* = 5) or type 2 diabetes (*n* = 4) from our analysis. Last, for repeated individual time-point analysis (i.e., not LME), we also limited the analysis to a subsample with all the measurements at all time-points to assess the robustness of results.

All statistical analyses were performed using the statistical software package STATA version 13.1 (StataCorp., College Station, TX, USA). A two-sided *p*-value < 0.05 was considered statistically significant.

## 3. Results

### 3.1. Maternal and Child Characteristics

Maternal and child characteristics according to quartiles of maternal dietary patterns score are shown in Table 2. Mothers who scored higher for the VFR pattern were older, more likely to be Chinese and to have a university degree; their children were less likely to be first-born (all *p* < 0.01). Mothers who scored higher for the SfN pattern were taller, had lower pre-pregnancy BMI, and more likely to be Chinese; their children were more likely to be first-born (all *p* ≤ 0.01). Lastly, more educated mothers scored higher for the PCB pattern (*p* = 0.012). There were also significant differences in maternal nutrient intake according to quartiles of maternal pattern scores (higher quartiles mean higher adherence to dietary pattern). For example, mothers in the highest quartile of VFR pattern had higher intakes of protein, total carbohydrate, starch, and dietary fiber but lower intakes of fat and sugar (all *p* ≤ 0.01), as compared with mothers in the lowest quartile.

### 3.2. Longitudinal Analysis (LME Models)

Table 3 shows the associations between maternal dietary patterns score and offspring indicators of adiposity from birth through 54 months assessed using LME models. In models adjusting for age at measurement only, a higher maternal VFR pattern score was associated with lower offspring BMI *z*-score and TS and higher offspring AC, but not with SS or SST. However, after adjusting for potential confounders (in particular ethnicity), the only significant association was that of higher VFR score with lower offspring TS (*β* = −0.09 mm per SD increase in VFR pattern score; 95% CI: −0.17 to −0.01 mm; *p* = 0.022). In contrast, higher maternal SfN pattern score was associated with higher offspring BMI *z*-score, SS, and AC (but not with TS and SST), but most of these associations were similarly attenuated after adjustment for confounders, in particular ethnicity. In the fully adjusted model, higher maternal SfN pattern score was only associated with higher offspring BMI *z*-score (*β* = 0.06 SD per SD increase in SfN pattern score; 95% CI: 0.01 to 0.12 SD; *p* = 0.026). The significant association between higher maternal VFR score and lower offspring TS thickness persisted following further adjustment for maternal smoking, alcohol intake, physical activities, and plasma vitamin D and folate concentrations (Supplementary Materials Table S3), and for postnatal factors including childhood diet, outdoor activity and media use (Supplementary Materials Table S4); the association between maternal SfN and offspring BMI *z*-score, however, was largely attenuated after these adjustments.

When small-for-gestational age, low birth weight, or total gestational weight gain was included in the LME models, the effect estimates (Supplementary Materials Table S5) remained largely similar as our main analysis, implying that our observed associations were unlikely to be mediated by these variables. Furthermore, when we excluded participants with preeclampsia and type 2 diabetes from our analysis, the results remained very similar. The PCB pattern was not associated with any of the offspring adiposity measures. There were no significant interactions between maternal dietary pattern and offspring sex and ethnicity for the assessed associations.

### 3.3. Individual Time-Points Analyses (Multiple Linear Regression Models)

Similar trends of associations were observed in the individual time-point analyses (Supplementary Materials Tables S6–S10). Overall, all of the derived patterns were not significantly associated with offspring adiposity measures at birth. However, a higher VFR pattern score tended to associate with lower offspring postnatal BMI *z*-score, although statistical significance in fully adjusted model was only observed at Months 18 and 48 (Supplementary Materials Table S6). Furthermore, a higher maternal VFR pattern score was consistently associated with lower offspring postnatal SS (*β* ranged from −0.17 to −0.31 mm; *p* < 0.05 at all postnatal time-points), TS (*β* ranged from −0.17 to −0.28 mm; *p* ≤ 0.01 at ages 18, 24, and 48 months), and SST (*β* ranged from −0.33 to −0.58 mm; *p* ≤ 0.05 at all postnatal time-points) (Supplementary Materials Tables S7–S9). In contrast, there were no clear associations between maternal SfN and PCB pattern scores and offspring adiposity, except that a higher maternal SfN score was generally associated with a higher offspring BMI *z*-score (Supplementary Materials Table S6). When we limited the individual time-point analyses to a subsample of children with anthropometric measurements at all time-points, the trends of associations between higher maternal VFR pattern score and lower offspring postnatal BMI *z*-score, SS, TS, and SST were similar (results not shown). However, for the association between maternal SfN pattern and childhood BMI *z*-score, no significant associations were observed at all time-points and the point estimates suggested a mix of direct and inverse associations at different time-points.

### 3.4. Associations between Quartiles of Maternal VFR Pattern Score and Childhood Adiposity (LME Models)

**Figure 2:**
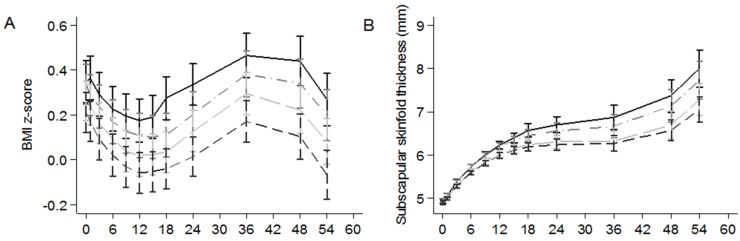

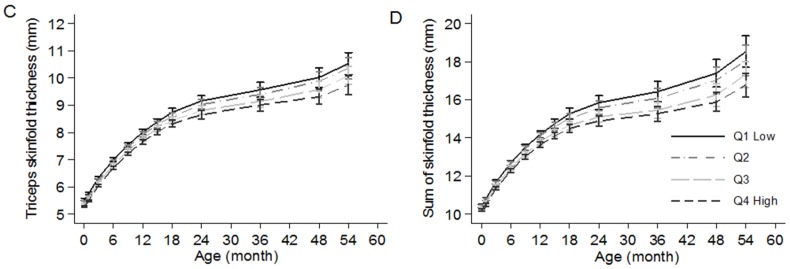
Predicted adjusted mean values with corresponding standard errors (from linear mixed effects models): (**A**) BMI *z*-score (*n* = 1048); (**B**) subscapular skinfold thickness (*n* = 1034); (**C**) triceps skinfold thickness (*n* = 1036); and (**D**) sum of skinfolds thickness (*n* = 1034) according to quartiles of maternal vegetables-fruit-and-white rice pattern score. The linear mixed effects models were adjusted for exact age at measurement, infant sex (except for BMI *z*-score), birth order, gestational age, duration of any breastfeeding, ethnicity, maternal age, height, pre-pregnancy BMI, weight gain until 26–28 weeks gestation, education level, gestational diabetes, energy intake, and scores of the other two dietary patterns (e.g., adjusting for SfN and PCB pattern scores for associations between VFR pattern and childhood adiposity). Median (range) of standardized dietary pattern scores: −1.09 (−3.26, −0.65) SD for Q1; −0.31 (−0.64, −0.02) SD for Q2; 0.28 (−0.02, 0.67) SD for Q3; 1.16 (0.68, 3.27) for Q4. Q, quartile.

Figure 2 shows that with increasing quartiles of maternal VFR pattern score, there are correspondingly lower predicted adjusted mean values (based on LME models) of offspring BMI *z*-score (Figure 2A), SS (Figure 2B), TS (Figure 2C), and SST (Figure 2D) from birth through 54 months of life. However, the differences in measurements of offspring adiposity across VFR quartiles only became more apparent from around 18 months of life, in concordance with our complementary individual time-point analyses.

## 4. Discussion

In this prospective Asian mother–offspring cohort study, we observed that a higher maternal VFR dietary pattern score was associated with overall lower offspring adiposity (especially at ≥18 months of age), indicated by lower BMI *z*-score, SS, TS, and SST. This suggests that greater adherence to the VFR pattern may be associated with lower offspring fat mass accretion. In contrast, maternal SfN and PCB dietary patterns were not consistently associated with offspring adiposity. To our knowledge, this is the first study that investigated the associations between maternal dietary patterns and offspring adiposity in an Asian population. Given the scarcity of evidence in this area, our study contributes to and extends the understanding of the potential influence of maternal diet on offspring adiposity measured at multiple time-points during infancy and early childhood.

Only two previous studies conducted in Ireland [15] and the Netherlands [16] have investigated the associations between maternal dietary patterns and offspring postnatal body composition or weight status. In the Irish study (*n* = 307), a higher maternal score on the “processed” pattern characterized by high intakes of soft drinks, chips, pizza, and sweets and chocolate was associated with a higher risk of offspring being overweight at five years of age. The result was consistent with our observation that a higher adherence to the VFR pattern characterized by lower intakes of Western fast food items (e.g., fried potatoes, carbonated drinks, and hamburger) was associated with lower offspring adiposity. In the Dutch study (*n* = 2695), two predominantly healthy dietary patterns characterized by: (1) vegetables, fish, and oil (VFO); and (2) nuts, soy, and high-fiber cereals (NSH), and an unhealthy pattern characterized by margarine, snacks, and sugar (MSS) were identified. In unadjusted analysis, higher maternal adherence to VFO and NSH patterns, but not MSS pattern, was associated with lower offspring BMI, fat mass index, and lower risk of being overweight at six years of life. However, the significant associations attenuated with adjustment for maternal and child sociodemographic and lifestyle factors, leading the authors to conclude that maternal dietary patterns were not independently associated with offspring body composition. However, in our study the associations between higher adherence to VFR pattern and lower offspring TS and SST were statistically significant even after adjustment for postnatal offspring diet at one year and outdoor activity at two years of age. The dietary patterns derived from our population and the Dutch population may not be directly comparable due to cultural differences. For instance, the NSH and MSS dietary patterns in the Dutch study were not identified in our study. Although our VFR pattern shares some similarity with their VFO pattern in terms of high positive loading of vegetables and negative loading of sugar-containing beverages, the two patterns differ in terms of fish and oil (high positive loading in the Dutch study) and fruits and white rice (high positive loading in our study). Furthermore, we have only included adiposity measures up to 4.5 years of age; subsequent follow up will allow us to investigate if later offspring adiposity is indeed less associated with maternal diet.

Several postulated mechanisms may underpin the association between greater maternal adherence to the VFR pattern and lower offspring adiposity. The VFR pattern is characterized by high consumption of fruit and vegetables and concurrently low intakes of Western fast food items. In terms of nutrient profile, greater adherence to the VFR pattern is associated with lower intake of sugar but higher intakes of protein and dietary fiber. A higher intake of vegetables and/or fruit has been associated with lower adiposity, weight gain, and lower overweight risk in both adult [46,47] and pediatric [48,49] populations, probably due partly to increased satiety and reduced energy density in a diet rich in fruit and vegetables, as they usually have low fat, high water, and high dietary fiber contents [50]. Furthermore, higher fast food intakes were associated with higher weight gain and higher risk of overweight, probably owing to the unfavorable nutrient profile (high fat, high sugar, and low micronutrients density) of fast food [51,52]. In addition, offspring of rat dams fed a high fat, high sugar diet during pregnancy showed a postnatal preference for fat, which might be explained by an altered development of the central mesolimbic reward system [53].

It is possible that our observed associations between greater maternal adherence to VFR pattern and lower offspring adiposity were due to transmission of eating and lifestyle habits within a family. However, our observed associations for VFR pattern persisted after further adjustment for offspring postnatal diet, outdoor activity, and media use. Although we acknowledge that residual confounding due to unmeasured and incompletely measured confounders may remain, it is likely that our observations were not purely attributable to shared family environment. Despite that, in order to disentangle the complex interplay between these factors, our results should be confirmed in further studies with more information on shared family diet (e.g., the availability of paternal diet and postnatal offspring diet at a later time point) and attendance of children in child care or preschool that might have affected their dietary patterns.

Alternatively, greater maternal adherence to a VFR pattern may have affected offspring adiposity through epigenetic mechanisms. For instance, offspring of pregnant dams fed a protein-restricted diet had decreased methylation status of their hepatic *PPARα* and *GR* genes, which in turn may be the underlying mechanism of impaired fat and carbohydrate metabolism and later hypertension induced by maternal protein restriction [54]. Similarly, in a *Drosophila* study, a high sugar maternal diet was associated with offspring metabolic defects (e.g., increased whole body glucose) that persisted in the F_2_ generation [55]. Finally, because higher maternal inflammation and oxidative stress have been associated with higher offspring adiposity in both human [56] and animal studies [57], a maternal dietary pattern rich in fruit and vegetables (and therefore rich in anti-oxidants [58,59]) may have resulted in lowered maternal inflammation and subsequently lowered offspring adiposity. It is not clear why the influence of maternal VFR pattern on offspring adiposity is more apparent during the later postnatal period (≥18 months of age) than at birth/during infancy in our study. Because adipose tissues accretion in the offspring starts only in late gestation and continues postnatally [60], it is possible that differences in adiposity become more prominent as the child grow. It may also be that maternal diet-induced alterations of gene expression in offspring, if any, tend to present themselves only later in life. For instance, in offspring of pregnant rats fed a protein-restricted diet, increased expression of *PPARα* and *CPT-1* (key genes in lipid and carbohydrate metabolism) was only detected in adulthood but not during neonatal period [61]. However, due to different physiological and metabolic properties in rodents and flies, the postulated mechanisms should be further explored and confirmed in a human population.

The prospective design of our study is important in establishing the temporal sequence for the association of maternal dietary pattern and offspring adiposity. Moreover, our frequent postnatal anthropometric and skinfold measurements increase the precision of offspring adiposity assessment. In addition, we characterized maternal diets using a dietary pattern approach which incorporates dietary behaviors and complex interactions among nutrients and food items. Furthermore, the longitudinal approach of our analysis elucidates the associations between maternal dietary pattern and offspring adiposity trajectory, taking into account within-person correlation of repeated measurements and missing outcome measurements.

However, our results should be interpreted in consideration of our study limitations. First, maternal dietary intake information in this study was assessed using a single 24-h recall, which may not provide adequate representation of an individual’s usual intake due to day-to-day variation in food intake. However, similar maternal dietary patterns have been extracted using data from 3-day food diary in a small subset of our study population (*n* = 212; [33]), suggesting that a single 24-h recall reasonably captured the usual maternal dietary patterns in our population. Second, assessment of maternal diet was done only once during late mid-gestation, and investigation of potential trimester-specific influence of maternal dietary pattern on offspring adiposity was not possible. However, it has been shown that dietary patterns change little during pregnancy [62,63], suggesting that dietary patterns derived at one time-point can be reasonably representative of the whole pregnancy period. Third, we did not collect information on paternal diet, which could have shed further insight on whether our observed associations were due to shared family environment or in utero fetal programming. Fourth, the non-response rate (787/2034 × 100% = 38.7%) in our cohort could potentially give rise to non-response bias. However, for this kind of bias to occur, the reason for non-participation must be related to both the exposure (maternal nutrition, data collected during 26th–28th week gestation) and outcome (offspring adiposity measures), which were both unknown during the time of recruitment (<14 weeks of gestation). Therefore, we do not expect that non-response bias significantly affected our results. Nonetheless, replication of results in other independent studies will increase the generalizability of our findings. Last, as in any observational study, residual confounding may have affected our observations and causality cannot be claimed.

## 5. Conclusions

In conclusion, in this multi-ethnic Asian mother–offspring cohort, a higher maternal VFR pattern score during pregnancy was associated with lower postnatal adiposity in the offspring. As the VFR pattern is characterized by high intakes of fruit and vegetables and low intakes of fast food items, promotion of such diets may reduce offspring adiposity. Although the observed effect sizes for reduced offspring adiposity may appear modest, childhood obesity is a multifactorial condition requiring an integrated strategy. Thus, pending confirmation from other independent studies and clinical trials, our results may point to potential new approaches to preventing childhood obesity.

## Figures and Tables

**Table 1:** Factor loadings for the maternal dietary patterns during pregnancy ^1^.

Food or Food Groups	VFR	SfN	PCB
Cruciferous, leafy-green and dark-yellow vegetables	0.52 *	-	-
Other vegetables ^2^	0.45 *	-	-
Fruits	0.37 *	-	-
White rice	0.31 *	−0.40	-
Non-fried red meat	0.26	-	-
Flavored rice ^3^	−0.27	-	-
Red meat and poultry (deep fried/in curry)	−0.29	-	-
Sweetened drinks ^4^	−0.29	-	-
Hamburger	−0.35	-	-
Carbonated drinks	−0.35	-	-
Fried potatoes	−0.44	-	-
Soup	-	0.46 *	-
Fish and seafood products	-	0.40 *	-
Flavored noodles ^5^	-	0.38 *	-
Noodles (in soup)	-	0.37 *	-
Non-fried red meat	-	0.37	-
Soya sauce based gravies	-	0.31	-
Seafood	-	0.29	-
Curry based gravies	-	−0.30	-
Legumes and pulses	-	−0.37	-
Ethnic bread ^6^	-	−0.44	-
Pasta	-	-	0.56 *
Tomato based gravies	-	-	0.56 *
Cheese	-	-	0.51 *
White bread	-	-	0.46 *
Margarine and peanut butter	-	-	0.32
Cream based gravies	-	-	0.31
Low fat milk	-	-	0.30
Whole-grain bread	-	-	0.26

^1^ Only food groups with loadings larger than 0.25 or smaller than −0.25 were shown. VFR, vegetables-fruit-and-white rice; SfN, seafood-and-noodles; PCB, pasta-cheese-and-bread; ^2^ Vegetables other than cruciferous, leafy-green and dark-yellow vegetables; ^3^ Chicken rice, nasi lemak, biryani, and flavored glutinous rice; ^4^ Non-carbonated, cordial and fruit drinks; ^5^ Stir-fried or gravy-based noodles such as char kway teow, Hokkien noodles, lor mee, and mee goreng; ^6^ Chinese steamed bun, tortilla, idli, puri, thosai, chapati, and naan; * Food items with top 4 positive loadings (unique to the dietary pattern) that were used to label the dietary patterns.

**Table 2:** Maternal and child characteristics according to quartiles of maternal dietary pattern scores ^1^.

	All (*n* = 1048)	Vegetables-Fruit-and-White Rice			Seafood-and-Noodles			Pasta-Cheese-and-Bread		
		Q1 (*n* = 262)	Q4 (*n* = 262)	*p*-Trend	Q1 (*n* = 262)	Q4 (*n* = 262)	*p*-Trend	Q1 (*n* = 261)	Q4 (*n* = 262)	*p*-Trend
**Maternal characteristics**										
Age, year	30.5 ± 5.1	28.4 ± 5.1	32.1 ± 4.8	**<0.001**	30.6 ± 5.3	30.9 ± 4.8	0.53	30.1 ± 5.0	30.8 ± 5.3	0.40
Height, cm	158.2 ± 5.6	157.7 ± 5.1	158.1 ± 5.8	0.35	157.4 ± 5.4	158.7 ± 5.5	**0.010**	157.8 ± 5.3	158.6 ± 5.6	0.19
Pre-pregnancy BMI, kg/m^2^	22.7 ± 4.4	22.8 ± 4.6	22.2 ± 3.7	0.07	23.4 ± 4.5	21.9 ± 4.0	**<0.001**	22.5 ± 4.1	22.7 ± 4.5	0.97
Weight gain till 26 weeks, kg	8.6 ± 4.4	8.9 ± 4.5	8.5 ± 4.0	0.12	8.2 ± 4.0	8.9 ± 4.2	0.09	8.8 ± 4.3	8.7 ± 4.3	0.91
Ethnicity				**<0.001**			**<0.001**			0.45
Chinese	580 (55.3%)	97 (37.0%)	197 (75.2%)		69 (26.3%)	211 (80.5%)		138 (52.9%)	153 (58.4%)	
Malay	275 (26.2%)	135 (51.5%)	18 (6.9%)		62 (23.7%)	45 (17.2%)		81 (31.0%)	74 (28.2%)	
Indian	193 (18.4%)	30 (11.5%)	47 (17.9%)		131 (50.0%)	6 (2.3%)		42 (16.1%)	35 (13.4%)	
Education status				**<0.001**			0.06			**0.012**
Primary/secondary	319 (30.4%)	94 (35.9%)	65 (24.8%)		69 (26.3%)	83 (31.7%)		99 (37.9%)	66 (25.2%)	
Post-secondary	384 (36.6%)	114 (43.5%)	81 (30.9%)		86 (32.8%)	94 (35.9%)		92 (35.3%)	106 (40.5%)	
University	345 (32.9%)	54 (20.6%)	116 (44.3%)		107 (40.8%)	85 (32.4%)		70 (26.8%)	90 (34.4%)	
**Maternal nutrient intake**										
Energy, kcal/day	1846 ± 562	1945 ± 578	1978 ± 530	0.37	1807 ± 576	2017 ± 515	**<0.001**	1878 ± 579	1997 ± 519	**<0.001**
Protein, % kcal/day	15.6 ± 3.8	14.9 ± 3.8	16.7 ± 4.1	**<0.001**	15.2 ±3.6	16.3 ± 3.8	**<0.001**	14.4 ± 3.6	17.2 ± 3.9	**<0.001**
Fat, % kcal/day	32.5 ± 7.6	35.2 ± 7.3	31.2 ± 7.7	**<0.001**	30.2 ± 8.0	33.9 ± 7.1	**<0.001**	33.5 ± 7.6	32.8 ± 6.9	0.63
Carbohydrate, % kcal/day	51.9 ± 8.8	49.9 ± 8.3	52.1 ± 9.4	**0.014**	54.7 ± 9.0	49.8 ± 8.1	**<0.001**	53.0 ± 9.3	50.0 ± 7.8	**0.001**
Sugar, % kcal/day	16.1 ± 7.1	17.2 ± 7.6	14.5 ± 6.3	**<0.001**	14.1 ± 6.5	16.6 ± 6.8	**<0.001**	16.9 ± 7.3	15.5 ± 6.2	**0.035**
Starch, % kcal/day	33.8 ± 9.6	33.9 ± 9.0	35.1 ± 10.2	**<0.001**	38.7 ± 10.6	32.2 ± 7.4	**<0.001**	33.2 ± 8.8	32.3 ± 8.5	0.07
Dietary fiber, g/1000 kcal	8.8 ± 4.3	7.2 ± 2.7	10.7 ± 5.2	**<0.001**	10.6 ± 6.2	8.3 ± 3.1	**<0.001**	8.4 ± 3.6	8.7 ± 4.4	0.89
**Child characteristics**										
Birth weight, kg	3.1 ± 0.5	3.0 ± 0.5	3.1 ± 0.5	0.36	3.1 ± 0.4	3.1 ± 0.5	0.06	3.1 ± 0.5	3.1 ± 0.5	0.20
Gestational age at birth, week	38.7 ± 1.4	38.6 ± 1.5	38.8 ± 1.4	0.48	38.8 ± 1.3	38.7 ± 1.3	0.39	38.7 ± 1.4	38.8 ± 1.5	0.24
Infant sex				0.32			0.10			0.60
Male	544 (51.9%)	131 (50.0%)	142 (54.2%)		125 (47.7%)	147 (56.1%)		130 (49.8%)	139 (53.1%)	
Female	504 (48.1%)	131 (50.0%)	120 (45.8%)		137 (52.3%)	115 (43.9%)		131 (50.2%)	123 (47.0%)	
Birth order				**0.004**			**0.014**			0.23
First-born	446 (42.6%)	128 (48.9%)	98 (37.4%)		94 (35.9%)	119 (45.4%)		119 (45.6%)	127 (48.5%)	
Non first-born	602 (57.4%)	134 (51.2%)	164 (62.6%)		168 (64.1%)	143 (54.6%)		142 (54.4%)	135 (51.5%)	

^1^ Values presented are mean ± SD or *n* (%). Q, quartile.

**Table 3:** Associations of maternal dietary pattern scores with indicators of offspring adiposity from birth through 54 months of age using linear mixed effects model.

	*n*	Vegetables-Fruit-and-White Rice		Seafood-and-Noodles		Pasta-Cheese-and-Bread	
		*β* (95% CI)	*p*	*β* (95% CI)	*p*	*β* (95% CI)	*p*
BMI *z*-score							
Model 1	1048	−0.06 (−0.11, −0.02) ^1^	**0.010**	0.06 (0.01, 0.11)	**0.012**	−0.01 (−0.05, 0.04)	0.84
Model 2	1048	−0.02 (−0.07, 0.03)	0.45	0.06 (0.01, 0.12)	**0.026**	−0.01 (−0.06, 0.03)	0.53
Subscapular skinfold, mm							
Model 1	1034	−0.04 (−0.10, 0.03)	0.25	0.06 (0.003, 0.12)	**0.039**	−0.001 (−0.06, 0.06)	0.97
Model 2	1034	−0.04 (−0.11, 0.02)	0.18	0.03 (−0.03, 0.10)	0.32	0.003 (−0.06, 0.06)	0.92
Triceps skinfold, mm							
Model 1	1036	−0.09 (−0.16, −0.02)	**0.008**	0.04 (−0.03, 0.10)	0.31	−0.01 (−0.08, 0.06)	0.82
Model 2	1036	−0.09 (−0.17, −0.01)	**0.022**	0.04 (−0.04, 0.12)	0.38	−0.004 (−0.07, 0.07)	0.90
Sum of skinfolds, mm							
Model 1	1034	−0.11 (−0.23, 0.01)	0.08	0.10 (−0.02, 0.22)	0.10	−0.01 (−0.13, 0.11)	0.85
Model 2	1034	−0.12 (−0.25, 0.01)	0.07	0.07 (−0.07, 0.21)	0.31	−0.003 (−0.12, 0.11)	0.96
Abdominal circumference, cm							
Model 1	1039	0.17 (0.05, 0.30)	**0.007**	0.20 (0.08, 0.33)	**0.002**	0.03 (−0.09, 0.16)	0.61
Model 2	1039	0.06 (−0.08, 0.19)	0.41	0.04 (−0.11, 0.18)	0.63	−0.02 (−0.14, 0.11)	0.80

^1^ All such values are β (95% CI) per 1 SD increment of maternal dietary pattern score. Model 1 adjusted for exact age at each measurement. Model 2 further adjusted for infant sex (except for BMI *z*-score), birth order, gestational age, duration of any breastfeeding, ethnicity, maternal age, height, pre-pregnancy BMI, weight gain until 26–28 weeks gestation, education level, gestational diabetes, energy intake, and scores of the other two dietary patterns (e.g., adjusting for SfN and PCB pattern scores for associations between VFR pattern and childhood adiposity.

## Supplementary Materials

The following are available online at http://www.mdpi.com/2072-6643/9/1/002/s1, Table S1. Complete list of the 68 food groups, Figure S1: Scree plot from exploratory factor analysis, Table S2: Inter-observer reliability of anthropometric measurements in the GUSTO study, Table S3: Associations of maternal dietary pattern scores with indicators of offspring adiposity from birth through 54 months of age using linear mixed effects model, further adjusting for maternal smoking, alcohol intake, physical activities, and plasma vitamin D and folate concentrations, Table S4: Associations of maternal dietary pattern scores with indicators of offspring adiposity from birth through 54 months of age using linear mixed effects model, further adjusting for childhood diet, outdoor activity, and media use, Table S5: Associations of maternal dietary pattern scores with indicators of offspring adiposity from birth through 54 months of age using linear mixed effects model, with (A) small-for-gestational age, (B) low birth weight, and (C) total gestational weight gain during pregnancy included as covariates to assess mediation effect, Table S6: Associations of maternal dietary patterns with offspring BMI *z*-score at specific time-points, Table S7: Associations of maternal dietary patterns with offspring subscapular skinfold thickness (mm) at specific time-points, Table S8: Associations of maternal dietary patterns with offspring triceps skinfold thickness (mm) at specific time-points, Table S9: Associations of maternal dietary patterns with offspring sum of skinfolds thickness (mm) at specific time-points, Table S10: Associations of maternal dietary patterns with offspring abdominal circumference (cm) at specific time-points.
