# Supplementary material for: Associations of Maternal Dietary Patterns during Pregnancy with Offspring Adiposity from Birth Until 54 Months of Age
Source: Nutrients. 2016 Dec 22;9(1):2. doi: 10.3390/nu9010002 (PMC5295046; doi:10.3390/nu9010002)
Supplement: Supplementary file 1 [file nutrients-09-00002-s001.docx]

**Supplementary Materials: Associations of Maternal Dietary Patterns during Pregnancy with Offspring Adiposity from Birth until 54 Months of Age**

Ling-Wei Chen, Izzuddin M. Aris, Jonathan Y. Bernard, Mya-Thway Tint, Airu Chia,
Marjorelee Colega, Peter D. Gluckman, Lynette Pei-Chi Shek, Seang-Mei Saw, Yap-Seng Chong, Fabian Yap, Keith M. Godfrey, Rob M. van Dam, Mary Foong-Fong Chong and Yung Seng Lee

**Table S1.** Complete list of the 68 food groups.

| **Food Groups** | |
| --- | --- |
| White rice | Grains |
| Brown rice | Oats |
| Flavoured rice | Breakfast cereals/bars |
| Porridge | Salad dressing |
| Noodles (in soup) | Sweet condiments |
| Flavoured noodles | Sweets |
| Pasta | Sweet spreads |
| White bread | Chocolate |
| Whole-grain bread | Low fat milk |
| Ethnic bread | Whole milk |
| Bread with toppings | Formula milk |
| Cruciferous, leafy-green and dark-yellow vegetables | Milk-based drinks |
| Other vegetables | Yogurt and cultured drinks |
| Fruits | Cheese |
| Fruit juice | Other dairy |
| Processed fruit | Carbonated drinks |
| Poultry (B) | Sweetened drinks |
| Red meat (B) | Soya bean drinks |
| Red meat and poultry (F) | Coffee and tea |
| Meat products | Dessert soup |
| Meat innards | Local sweet snacks/pastries/biscuits |
| Fish (B) | Local savoury snacks (B) |
| Fish (F) | Local savoury snacks (F) |
| Seafood | Ice-cream |
| Fish and seafood products | Chips |
| Eggs | Hamburger |
| Beancurd | Pizza |
| Legumes and pulses | Fried potatoes |
| Nuts and seeds | Blended oil |
| Tomato based gravies | Monounsaturated and polyunsaturated oils |
| Cream based gravies | Butter and ghee |
| Curry based gravies | Margarine and peanut butter |
| Soya sauce based gravies | Soup |
| Other gravies | Cream-based soup |

F = deep-fried or cooked in curry; B = boiled, steamed, grilled, roasted, baked, stir fried, braised, or stewed preparation.

**Figure S1.** Scree plot from exploratory factor analysis. VFR: vegetables-fruit-and-white rice; SfN: seafood-and-noodles; PCB: pasta-cheese-and-bread.

**Table S2.** Inter-observer reliability of anthropometric measurements in the GUSTO study.

|  | **TEM** | **CV (%)** |
| --- | --- | --- |
| Weight, kg | 0.09 | 0.19 |
| Length, cm | 0.26 | 0.16 |
| Abdominal circumference, cm | 0.30 | 0.50 |
| Triceps skinfold, mm | 0.80 | 6.00 |
| Subscapular skinfold, mm | 0.83 | 4.18 |

TEM, technical error of measurement; CV, coefficient of variation.

**Table S3.** Associations of maternal dietary pattern scores with indicators of offspring adiposity from birth through 54 months of age using linear mixed effects model, further adjusting for maternal smoking, alcohol intake, physical activities, and plasma vitamin D and folate concentrations.

|  | *n* | Vegetables-Fruit-and-White Rice | | Seafood-and-Noodles | | Pasta-Cheese-and-Bread | |
| --- | --- | --- | --- | --- | --- | --- | --- |
|  |  | *β* (95% CI) | *p* | *β* (95% CI) | *p* | *β* (95% CI) | *p* |
| BMI *z*-score | 920 | −0.03 (−0.09, 0.02) ^1^ | 0.23 | 0.05 (−0.01, 0.11) | 0.07 | −0.01 (−0.06, 0.04) | 0.81 |
| Subscapular skinfold | 913 | −0.05 (−0.11, 0.02) | 0.19 | 0.04 (−0.03, 0.11) | 0.31 | −0.01 (−0.07, 0.06) | 0.83 |
| Triceps skinfold | 915 | −0.11 (−0.19, −0.03) | 0.005 | 0.02 (−0.06, 0.11) | 0.58 | −0.01 (−0.08, 0.06) | 0.77 |
| Sum of skinfolds | 913 | −0.15 (−0.28, −0.01) | 0.031 | 0.06 (−0.08, 0.21) | 0.39 | −0.02 (−0.15, 0.10) | 0.72 |
| Abdominal circumference | 918 | 0.06 (−0.09, 0.20) | 0.45 | 0.04 (−0.12, 0.19) | 0.65 | −0.01 (−0.14, 0.12) | 0.82 |

^1^ All such values are *β* (95% CI) per 1 SD increment of maternal dietary pattern score. Model adjusted for exact age at each measurement, infant sex (except for BMI *z*-score), birth order, gestational age, duration of any breastfeeding, ethnicity, maternal age, height, pre-pregnancy BMI, weight gain until 26–28 weeks gestation, education level, gestational diabetes, energy intake, scores of the other two dietary patterns (e.g., adjusting for SfN and PCB pattern scores for associations between VFR pattern and childhood adiposity), and maternal smoking, alcohol intake, physical activities, and plasma vitamin D and folate concentrations.

**Table S4.** Associations of maternal dietary pattern scores with indicators of offspring adiposity from birth through 54 months of age using linear mixed effects model, further adjusting for childhood diet, outdoor activity, and media use.

|  | ***n*** | **Vegetables-Fruit-and-White Rice** | | **Seafood-and-Noodles** | | **Pasta-Cheese-and-Bread** | |
| --- | --- | --- | --- | --- | --- | --- | --- |
|  |  | ***β* (95% CI)** | ***p*** | ***β* (95% CI)** | ***p*** | ***β* (95% CI)** | ***p*** |
| BMI *z*-score | 656 | −0.04 (−0.10, 0.02) ^1^ | 0.18 | 0.02 (−0.05, 0.08) | 0.56 | 0.02 (−0.04, 0.08) | 0.53 |
| Subscapular skinfold | 656 | −0.07 (−0.15, 0.01) | 0.08 | 0.03 (−0.05, 0.12) | 0.42 | 0.02 (−0.05, 0.10) | 0.55 |
| Triceps skinfold | 656 | **−0.15 (−0.24, −0.05)** | **0.002** | 0.03 (−0.07, 0.12) | 0.62 | 0.03 (−0.06, 0.12) | 0.55 |
| Sum of skinfolds | 656 | **−0.20 (−0.36, −0.05)** | **0.011** | 0.07 (−0.10, 0.24) | 0.43 | 0.05 (−0.10, 0.20) | 0.52 |
| Abdominal circumference | 656 | −0.01 (−0.17, 0.15) | 0.91 | 0.03 (−0.14, 0.20) | 0.75 | 0.13 (−0.03, 0.28) | 0.11 |

^1^ All such values are *β* (95% CI) per 1 SD increment of maternal dietary pattern score. Model adjusted for exact age at each measurement, infant sex (except for BMI *z*-score), birth order, gestational age, duration of any breastfeeding, ethnicity, maternal age, height, pre-pregnancy BMI, weight gain until 26–28 weeks gestation, education level, gestational diabetes, energy intake, scores of the other two dietary patterns (e.g., adjusting for SfN and PCB pattern scores for associations between VFR pattern and childhood adiposity) and childhood diet, outdoor activity, and media use.

**Table S5.** Associations of maternal dietary pattern scores with indicators of offspring adiposity from birth through 54 months of age using linear mixed effects model, with (A) small-for-gestational age, (B) low birth weight, and (C) total gestational weight gain during pregnancy included as covariates to assess mediation effect.

| **(A) Small-for-Gestational Age** | | | | | | | |
| --- | --- | --- | --- | --- | --- | --- | --- |
|  | ***n*** | **Vegetables-Fruit-and-White Rice** | | **Seafood-and-Noodles** | | **Pasta-Cheese-and-Bread** | |
|  |  | ***β* (95% CI)** | ***p*** | ***β* (95% CI)** | ***p*** | ***β* (95% CI)** | ***p*** |
| BMI *z*-score | 1048 | −0.01 (−0.06, 0.04) ^1^ | 0.58 | **0.07 (0.02, 0.12)** | **0.010** | −0.01 (−0.06, 0.03) | 0.58 |
| Subscapular skinfold | 1034 | −0.04 (−0.10, 0.03) | 0.25 | 0.05 (−0.02, 0.11) | 0.19 | 0.004 (−0.05, 0.06) | 0.89 |
| Triceps skinfold | 1036 | **−0.08 (−0.16, −0.01)** | **0.029** | 0.05 (−0.03, 0.13) | 0.26 | −0.003 (−0.07, 0.07) | 0.94 |
| Sum of skinfolds | 1034 | −0.11 (−0.23, 0.02) | 0.10 | 0.09 (−0.04, 0.23) | 0.18 | −0.0004 (−0.12, 0.11) | 1.00 |
| Abdominal circumference | 1039 | 0.07 (−0.06, 0.20) | 0.28 | 0.06 (−0.08, 0.20) | 0.38 | −0.004 (−0.12, 0.11) | 0.95 |
| **(B) Low Birth Weight** | | | | | | | |
|  | ***n*** | **Vegetables-Fruit-and-White Rice** | | **Seafood-and-Noodles** | | **Pasta-Cheese-and-Bread** | |
|  |  | ***β* (95% CI)** | ***p*** | ***β* (95% CI)** | ***p*** | ***β* (95% CI)** | ***p*** |
| BMI *z*-score | 1048 | −0.02 (−0.07, 0.03) | 0.46 | **0.06 (0.01, 0.12)** | **0.022** | −0.01 (−0.06, 0.03) | 0.54 |
| Subscapular skinfold | 1034 | −0.04 (−0.11, 0.02) | 0.20 | 0.04 (−0.03, 0.11) | 0.28 | 0.004 (−0.05, 0.06) | 0.89 |
| Triceps skinfold | 1036 | **−0.09 (−0.16, −0.01)** | **0.023** | 0.04 (−0.04, 0.12) | 0.34 | −0.003 (−0.07, 0.07) | 0.93 |
| Sum of skinfolds | 1034 | −0.11 (−0.24, 0.01) | 0.08 | 0.08 (−0.06, 0.21) | 0.27 | −0.001 (−0.12, 0.12) | 0.99 |
| Abdominal circumference | 1039 | 0.06 (−0.07, 0.19) | 0.38 | 0.04 (−0.10, 0.18) | 0.57 | −0.01 (−0.13, 0.11) | 0.85 |
| **(C) Total gestational weight gain** | | | | | | | |
|  | ***n*** | **Vegetables-Fruit-and-White Rice** | | **Seafood-and-Noodles** | | **Pasta-Cheese-and-Bread** | |
|  |  | ***β* (95% CI)** | ***p*** | ***β* (95% CI)** | ***p*** | ***β* (95% CI)** | ***p*** |
| BMI *z*-score | 1048 | −0.02 (−0.07, 0.03) | 0.37 | **0.06 (0.01, 0.11)** | **0.032** | −0.01 (−0.06, 0.03) | 0.53 |
| Subscapular skinfold | 1034 | −0.05 (−0.11, 0.02) | 0.14 | 0.03 (−0.04, 0.10) | 0.36 | 0.002 (−0.06, 0.06) | 0.94 |
| Triceps skinfold | 1036 | **−0.09 (−0.17, −0.02)** | **0.015** | 0.03 (−0.05, 0.11) | 0.43 | −0.01 (−0.07, 0.06) | 0.89 |
| Sum of skinfolds | 1034 | −0.13 (−0.26, 0.001) | 0.05 | 0.06 (−0.07, 0.20) | 0.36 | −0.004 (−0.12, 0.11) | 0.94 |
| Abdominal circumference | 1039 | 0.05 (−0.09, 0.18) | 0.49 | 0.03 (−0.12, 0.17) | 0.72 | −0.02 (−0.14, 0.10) | 0.79 |

^1^ All such values are *β* (95% CI) per 1 SD increment of maternal dietary pattern score. Model adjusted for exact age at each measurement, infant sex (except for BMI *z*-score), birth order, gestational age, duration of any breastfeeding, ethnicity, maternal age, height, pre-pregnancy BMI, weight gain until 26–28 weeks gestation, education level, gestational diabetes, energy intake, scores of the other two dietary patterns (e.g., adjusting for SfN and PCB pattern scores for associations between VFR pattern and childhood adiposity). Furthermore, small-for-gestational age, low birth weight, and total gestational weight gain were included as covariates in Table S5A–C, respectively, to assess potential mediation effects. For mediation model with total gestational weight gain, weight gain until 26–28 weeks gestation was removed from the model.

**Table S6.** Associations of maternal dietary patterns with offspring BMI *z*-score at specific time-points.

|  | ***n*** | **Vegetables-Fruit-and-White Rice** | | **Seafood-and-Noodles** | | **Pasta-Cheese-and-Bread** | |
| --- | --- | --- | --- | --- | --- | --- | --- |
|  |  | ***β* (95% CI)** | ***p*** | ***β* (95% CI)** | ***p*** | ***β* (95% CI)** | ***p*** |
| BMI at birth |  |  |  |  |  |  |  |
| Model 1 | 1048 | 0.02 (−0.05, 0.09) ^1^ | 0.52 | **0.07 (0.004, 0.14)** | **0.039** | 0.001 (−0.07, 0.07) | 0.97 |
| Model 2 | 1048 | 0.07 (−0.002, 0.13) | 0.06 | 0.07 (−0.001, 0.15) | 0.05 | 0.002 (−0.06, 0.06) | 0.95 |
| BMI at 3 months |  |  |  |  |  |  |  |
| Model 1 | 922 | −0.03 (−0.10, 0.03) | 0.32 | **0.14 (0.07, 0.20)** | **<0.001** | −0.01 (−0.08, 0.06) | 0.81 |
| Model 2 | 922 | −0.02 (−0.10, 0.05) | 0.52 | −0.003 (−0.08, 0.08) | 0.95 | −0.02 (−0.09, 0.04) | 0.48 |
| BMI at 6 months |  |  |  |  |  |  |  |
| Model 1 | 886 | −0.07 (−0.14, 0.01) | 0.08 | **0.09 (0.02, 0.16)** | **0.013** | 0.01 (−0.07, 0.08) | 0.90 |
| Model 2 | 886 | −0.02 (−0.10, 0.06) | 0.60 | 0.03 (−0.05, 0.12) | 0.48 | −0.02 (−0.09, 0.06) | 0.67 |
| BMI at 9 months |  |  |  |  |  |  |  |
| Model 1 | 845 | **−0.10 (−0.18, −0.03)** | **0.006** | 0.03 (−0.04, 0.10) | 0.43 | −0.01 (−0.09, 0.06) | 0.69 |
| Model 2 | 845 | −0.06 (−0.14, 0.02) | 0.17 | 0.03 (−0.05, 0.12) | 0.47 | −0.03 (−0.10, 0.04) | 0.39 |
| BMI at 12 months |  |  |  |  |  |  |  |
| Model 1 | 861 | **−0.10 (−0.17, −0.03)** | **0.004** | 0.06 (−0.01, 0.13) | 0.08 | −0.02 (−0.09, 0.05) | 0.62 |
| Model 2 | 861 | −0.05 (−0.13, 0.03) | 0.21 | **0.09 (0.01, 0.17)** | **0.028** | −0.02 (−0.10, 0.05) | 0.50 |
| BMI at 15 months |  |  |  |  |  |  |  |
| Model 1 | 860 | **−0.13 (−0.20, −0.06)** | **<0.001** | 0.07 (−0.001, 0.14) | 0.05 | 0.01 (−0.06, 0.08) | 0.77 |
| Model 2 | 860 | −0.07 (−0.15, 0.01) | 0.07 | **0.10 (0.01, 0.18)** | **0.021** | −0.001 (−0.07, 0.07) | 0.98 |
| BMI at 18 months |  |  |  |  |  |  |  |
| Model 1 | 718 | **−0.13 (−0.20, −0.05)** | **0.001** | 0.05 (−0.01, 0.12) | 0.12 | 0.03 (−0.04, 0.11) | 0.39 |
| Model 2 | 718 | **−0.10 (−0.18, −0.02)** | **0.013** | **0.09 (0.002, 0.17)** | **0.045** | 0.02 (−0.05, 0.09) | 0.60 |
| BMI at 24 months |  |  |  |  |  |  |  |
| Model 1 | 723 | **−0.11 (−0.18, −0.04)** | **0.004** | 0.06 (−0.01, 0.13) | 0.09 | 0.03 (−0.05, 0.10) | 0.50 |
| Model 2 | 723 | −0.07 (−0.15, 0.01) | 0.08 | **0.08 (0.001, 0.17)** | **0.048** | 0.01 (−0.07, 0.08) | 0.86 |
| BMI at 36 months |  |  |  |  |  |  |  |
| Model 1 | 834 | **−0.09 (−0.16, −0.03)** | **0.003** | 0.04 (−0.02, 0.10) | 0.16 | −0.03 (−0.09, 0.03) | 0.35 |
| Model 2 | 834 | −0.04 (−0.11, 0.02) | 0.20 | 0.07 (−0.004, 0.14) | 0.07 | −0.03 (−0.09, 0.03) | 0.33 |
| BMI at 48 months |  |  |  |  |  |  |  |
| Model 1 | 773 | **−0.12 (−0.18, −0.06)** | **<0.001** | 0.02 (−0.04, 0.07) | 0.62 | 0.01 (−0.05, 0.08) | 0.73 |
| Model 2 | 773 | **−0.08 (−0.14, −0.01)** | **0.027** | 0.02 (−0.04, 0.09) | 0.48 | 0.01 (−0.05, 0.07) | 0.77 |
| BMI at 54 months |  |  |  |  |  |  |  |
| Model 1 | 763 | **−0.11 (−0.17, −0.04)** | **0.001** | 0.01 (−0.05, 0.07) | 0.77 | 0.01 (−0.06, 0.08) | 0.78 |
| Model 2 | 763 | −0.06 (−0.13, 0.01) | 0.08 | 0.04 (−0.03, 0.11) | 0.27 | 0.01 (−0.06, 0.07) | 0.81 |

^1^ All such values are *β* (95% CI) per 1 SD increment of maternal dietary pattern score. Model 1 adjusted for exact age at each measurement. Model 2 further adjusted for birth order, gestational age, duration of any breastfeeding, ethnicity, maternal age, height, pre-pregnancy BMI, weight gain until 26–28 weeks gestation, education level, gestational diabetes, energy intake, and scores of the other two dietary patterns (e.g., adjusting for SfN and PCB patterns score for associations between VFR pattern and childhood adiposity).

**Table S7.** Associations of maternal dietary patterns with offspring subscapular skinfold thickness (mm) at specific time-points.

|  | ***n*** | **Vegetables-Fruit-and-White Rice** | | **Seafood-and-Noodles** | | **Pasta-Cheese-and-Bread** | |
| --- | --- | --- | --- | --- | --- | --- | --- |
|  |  | ***β* (95% CI)** | ***p*** | ***β* (95% CI)** | ***p*** | ***β* (95% CI)** | ***p*** |
| SS at birth |  |  |  |  |  |  |  |
| Model 1 | 998 | 0.05 (−0.02, 0.13) ^1^ | 0.14 | 0.03 (−0.04, 0.10) | 0.42 | −0.03 (−0.11, 0.04) | 0.37 |
| Model 2 | 998 | 0.07 (−0.01, 0.15) | 0.07 | 0.01 (−0.07, 0.09) | 0.83 | −0.02 (−0.09, 0.05) | 0.54 |
| SS at 18 months |  |  |  |  |  |  |  |
| Model 1 | 692 | **−0.16 (−0.27, −0.06)** | **0.002** | 0.08 (−0.02, 0.18) | 0.14 | 0.08 (−0.03, 0.18) | 0.15 |
| Model 2 | 692 | **−0.19 (−0.30, −0.08)** | **0.001** | 0.10 (−0.02, 0.22) | 0.12 | 0.09 (−0.02, 0.19) | 0.11 |
| SS at 24 months |  |  |  |  |  |  |  |
| Model 1 | 775 | **−0.19 (−0.30, −0.08)** | **0.001** | 0.04 (−0.07, 0.15) | 0.43 | 0.08 (−0.04, 0.20) | 0.20 |
| Model 2 | 775 | **−0.21 (−0.33, −0.08)** | **0.001** | 0.06 (−0.07, 0.19) | 0.38 | 0.09 (−0.03, 0.20) | 0.16 |
| SS at 36 months |  |  |  |  |  |  |  |
| Model 1 | 811 | **−0.17 (−0.32, −0.03)** | **0.016** | 0.06 (−0.08, 0.21) | 0.38 | −0.0001 (−0.15, 0.15) | 1.00 |
| Model 2 | 811 | **−0.17 (−0.32, −0.01)** | **0.035** | 0.05 (−0.11, 0.22) | 0.53 | 0.01 (−0.13, 0.16) | 0.85 |
| SS at 48 months |  |  |  |  |  |  |  |
| Model 1 | 727 | **−0.36 (−0.56, −0.16)** | **<0.001** | −0.03 (−0.22, 0.16) | 0.75 | 0.07 (−0.13, 0.27) | 0.49 |
| Model 2 | 727 | **−0.31 (−0.52, −0.09)** | **0.005** | 0.08 (−0.14, 0.30) | 0.46 | 0.12 (−0.08, 0.31) | 0.24 |
| SS at 54 months |  |  |  |  |  |  |  |
| Model 1 | 741 | **−0.37 (−0.61, −0.12)** | **0.004** | −0.13 (−0.36, 0.11) | 0.29 | 0.16 (−0.10, 0.41) | 0.23 |
| Model 2 | 741 | **−0.30 (−0.56, −0.03)** | **0.027** | 0.16 (−0.11, 0.43) | 0.26 | 0.20 (−0.04, 0.45) | 0.11 |

^1^ All such values are *β* (95% CI) per 1 SD increment of maternal dietary pattern score. SS, subscapular skinfold Model 1 adjusted for exact age at each measurement. Model 2 further adjusted for infant sex, birth order, gestational age, duration of any breastfeeding, ethnicity, maternal age, height, pre-pregnancy BMI, weight gain until 26–28 weeks gestation, education level, gestational diabetes, energy intake, and scores of the other two dietary patterns (e.g., adjusting for SfN and PCB pattern scores for associations between VFR pattern and childhood adiposity).

**Table S8.** Associations of maternal dietary patterns with offspring triceps skinfold thickness (mm) at specific time-points.

|  | ***n*** | **Vegetables-Fruit-and-White Rice** | | **Seafood-and-Noodles** | | **Pasta-Cheese-and-Bread** | |
| --- | --- | --- | --- | --- | --- | --- | --- |
|  |  | ***β* (95% CI)** | ***p*** | ***β* (95% CI)** | ***p*** | ***β* (95% CI)** | ***p*** |
| TS at birth |  |  |  |  |  |  |  |
| Model 1 | 999 | 0.02 (−0.06, 0.10) ^1^ | 0.65 | 0.04 (−0.04, 0.11) | 0.35 | −0.02 (−0.10, 0.06) | 0.60 |
| Model 2 | 999 | 0.02 (−0.06, 0.11) | 0.62 | 0.01 (−0.08, 0.10) | 0.79 | −0.01 (−0.09, 0.06) | 0.71 |
| TS at 18 months |  |  |  |  |  |  |  |
| Model 1 | 737 | **−0.26 (−0.39, −0.13)** | **<0.001** | −0.02 (−0.14, 0.11) | 0.81 | 0.08 (−0.05, 0.21) | 0.23 |
| Model 2 | 737 | **−0.23 (−0.37, −0.08)** | **0.002** | 0.02 (−0.13, 0.17) | 0.77 | 0.09 (−0.04, 0.22) | 0.17 |
| TS at 24 months |  |  |  |  |  |  |  |
| Model 1 | 754 | **−0.23 (−0.37, −0.09)** | **0.001** | −0.01 (−0.15, 0.12) | 0.84 | −0.01 (−0.15, 0.14) | 0.95 |
| Model 2 | 754 | **−0.20 (−0.36, −0.05)** | **0.010** | 0.05 (−0.11, 0.22) | 0.51 | 0.002 (−0.15, 0.15) | 0.98 |
| TS at 36 months |  |  |  |  |  |  |  |
| Model 1 | 803 | **−0.19 (−0.35, −0.03)** | **0.020** | 0.10 (−0.06, 0.27) | 0.20 | −0.08 (−0.25, 0.09) | 0.34 |
| Model 2 | 803 | −0.17 (−0.35, 0.01) | 0.06 | **0.20 (0.08, 0.39)** | **0.041** | −0.06 (−0.22, 0.11) | 0.51 |
| TS at 48 months |  |  |  |  |  |  |  |
| Model 1 | 737 | **−0.32 (−0.53, −0.11)** | **0.002** | −0.01 (−0.21, 0.18) | 0.90 | 0.12 (−0.09, 0.33) | 0.27 |
| Model 2 | 737 | **−0.28 (−0.51, −0.06)** | **0.013** | 0.10 (−0.13, 0.33) | 0.39 | 0.16 (−0.04, 0.36) | 0.11 |
| TS at 54 months |  |  |  |  |  |  |  |
| Model 1 | 740 | −0.21 (−0.44, 0.02) | 0.07 | −0.14 (−0.36, 0.08) | 0.22 | 0.09 (−0.15, 0.33) | 0.47 |
| Model 2 | 740 | −0.18 (−0.42, 0.07) | 0.16 | 0.11 (−0.14, 0.36) | 0.39 | 0.10 (−0.13, 0.32) | 0.41 |

^1^ All such values are *β* (95% CI) per 1 SD increment of maternal dietary pattern score.TS, triceps skinfold Model 1 adjusted for exact age at measurement. Model 2 further adjusted for infant sex, birth order, gestational age, duration of any breastfeeding, ethnicity, maternal age, height, pre-pregnancy BMI, weight gain until 26–28 weeks gestation, education level, gestational diabetes, energy intake, and scores of the other two dietary patterns (e.g., adjusting for SfN and PCB pattern scores for associations between VFR pattern and childhood adiposity).

**Table S9.** Associations of maternal dietary patterns with offspring sum of skinfolds thickness (mm) at specific time-points.

|  | ***n*** | **Vegetables-Fruit-and-White Rice** | | **Seafood-and-Noodles** | | **Pasta-Cheese-and-Bread** | |
| --- | --- | --- | --- | --- | --- | --- | --- |
|  |  | ***β* (95% CI)** | ***p*** | ***β* (95% CI)** | ***p*** | ***β* (95% CI)** | ***p*** |
| SST at birth |  |  |  |  |  |  |  |
| Model 1 | 998 | 0.07 (−0.07, 0.21) ^1^ | 0.31 | 0.07 (−0.07, 0.20) | 0.34 | −0.05 (−0.19, 0.08) | 0.44 |
| Model 2 | 998 | 0.09 (−0.05, 0.24) | 0.22 | 0.02 (−0.14, 0.18) | 0.80 | −0.04 (−0.17, 0.10) | 0.59 |
| SST at 18 months |  |  |  |  |  |  |  |
| Model 1 | 667 | **−0.40 (−0.62, −0.18)** | **<0.001** | 0.06 (−0.15, 0.27) | 0.59 | 0.16 (−0.06, 0.38) | 0.15 |
| Model 2 | 667 | **−0.41 (−0.65, −0.17)** | **0.001** | 0.12 (−0.13, 0.38) | 0.34 | 0.18 (−0.03, 0.40) | 0.10 |
| SST at 24 months |  |  |  |  |  |  |  |
| Model 1 | 740 | **−0.39 (−0.63, −0.16)** | **0.001** | 0.04 (−0.18, 0.27) | 0.72 | 0.08 (−0.17, 0.32) | 0.55 |
| Model 2 | 740 | **−0.38 (−0.64, −0.12)** | **0.004** | 0.16 (−0.11, 0.43) | 0.26 | 0.09 (−0.16, 0.33) | 0.49 |
| SST at 36 months |  |  |  |  |  |  |  |
| Model 1 | 797 | **−0.37 (−0.65, −0.08)** | **0.012** | 0.16 (−0.12, 0.45) | 0.26 | −0.10 (−0.40, 0.20) | 0.53 |
| Model 2 | 797 | **−0.33 (−0.64, −0.03)** | **0.034** | 0.25 (−0.09, 0.58) | 0.15 | −0.05 (−0.35, 0.24) | 0.72 |
| SST at 48 months |  |  |  |  |  |  |  |
| Model 1 | 725 | **−0.68 (−1.07, −0.29)** | **0.001** | −0.03 (−0.40, 0.33) | 0.86 | 0.18 (−0.21, 0.57) | 0.37 |
| Model 2 | 725 | **−0.58 (−1.00, −0.16)** | **0.007** | 0.20 (−0.23, 0.62) | 0.37 | 0.26 (−0.11, 0.64) | 0.17 |
| SST at 54 months |  |  |  |  |  |  |  |
| Model 1 | 738 | **−0.59 (−1.04, −0.14)** | **0.011** | −0.27 (−0.70, 0.17) | 0.23 | 0.24 (−0.22, 0.71) | 0.31 |
| Model 2 | 738 | −0.48 (−0.95, 0.002) | 0.05 | 0.23 (−0.26, 0.72) | 0.35 | 0.29 (−0.15, 0.73) | 0.20 |

^1^ All such values are *β* (95% CI) per 1 SD increment of maternal dietary pattern score. SST, sum of skinfolds thickness. Model 1 adjusted for exact age at each measurement. Model 2 further adjusted for infant sex, birth order, gestational age, duration of any breastfeeding, ethnicity, maternal age, height, pre-pregnancy BMI, weight gain until 26–28 weeks gestation, education level, gestational diabetes, energy intake, and scores of the other two dietary patterns (e.g., adjusting for SfN and PCB pattern scores for associations between VFR pattern and childhood adiposity).

**Table S10.** Associations of maternal dietary patterns with offspring abdominal circumference (cm) at specific time-points.

|  | ***n*** | **Vegetables-Fruit-and-White Rice** | | **Seafood-and-Noodles** | | **Pasta-Cheese-and-Bread** | |
| --- | --- | --- | --- | --- | --- | --- | --- |
|  |  | ***β* (95% CI)** | ***p*** | **Β (95% CI)** | ***p*** | ***β* (95% CI)** | ***p*** |
| AC at birth |  |  |  |  |  |  |  |
| Model 1 | 997 | −0.01 (−0.16, 0.14) ^1^ | 0.91 | 0.03 (−0.12, 0.19) | 0.65 | 0.12 (−0.03, 0.27) | 0.12 |
| Model 2 | 997 | 0.08 (−0.08, 0.24) | 0.32 | −0.02 (−0.20, 0.15) | 0.81 | 0.10 (−0.05, 0.24) | 0.20 |
| AC at 3 months |  |  |  |  |  |  |  |
| Model 1 | 920 | **0.23 (0.04, 0.43)** | **0.020** | **0.48 (0.29, 0.67)** | **<0.001** | −0.12 (−0.32, 0.08) | 0.23 |
| Model 2 | 920 | 0.04 (−0.16, 0.25) | 0.68 | 0.14 (−0.09, 0.36) | 0.24 | −0.16 (−0.35, 0.03) | 0.10 |
| AC at 6 months |  |  |  |  |  |  |  |
| Model 1 | 884 | **0.23 (0.02, 0.44)** | **0.032** | 0.18 (−0.03, 0.38) | 0.09 | 0.05 (−0.16, 0.25) | 0.67 |
| Model 2 | 884 | 0.11 (−0.12, 0.34) | 0.33 | −0.05 (−0.30, 0.19) | 0.66 | 0.01 (−0.20, 0.21) | 0.96 |
| AC at 9 months |  |  |  |  |  |  |  |
| Model 1 | 844 | 0.16 (−0.05, 0.38) | 0.14 | 0.20 (−0.01, 0.41) | 0.06 | 0.19 (−0.02, 0.40) | 0.08 |
| Model 2 | 844 | 0.02 (−0.22, 0.26) | 0.86 | 0.01 (−0.24, 0.26) | 0.96 | 0.14 (−0.07, 0.35) | 0.19 |
| AC at 12 months |  |  |  |  |  |  |  |
| Model 1 | 866 | **0.28 (0.07, 0.50)** | **0.010** | 0.19 (−0.01, 0.40) | 0.07 | 0.09 (−0.13, 0.31) | 0.41 |
| Model 2 | 866 | 0.16 (−0.08, 0.40) | 0.18 | 0.10 (−0.15, 0.35) | 0.44 | 0.07 (−0.15, 0.29) | 0.52 |
| AC at 15 months |  |  |  |  |  |  |  |
| Model 1 | 867 | 0.07 (−0.14, 0.28) | 0.52 | 0.07 (−0.13, 0.28) | 0.49 | 0.07 (−0.14, 0.28) | 0.52 |
| Model 2 | 867 | −0.09 (−0.32, 0.14) | 0.43 | −0.06 (−0.30, 0.18) | 0.62 | 0.02 (−0.19, 0.23) | 0.84 |
| AC at 18 months |  |  |  |  |  |  |  |
| Model 1 | 784 | −0.07 (−0.30, 0.16) | 0.54 | −0.02 (−0.23, 0.20) | 0.89 | 0.18 (−0.05, 0.42) | 0.12 |
| Model 2 | 784 | −0.13 (−0.39, 0.12) | 0.29 | 0.06 (−0.20, 0.33) | 0.64 | 0.18 (−0.05, 0.42) | 0.12 |
| AC at 24 months |  |  |  |  |  |  |  |
| Model 1 | 795 | −0.0001 (−0.24, 0.24) | 1.00 | 0.08 (−0.15, 0.32) | 0.48 | 0.14 (−0.12, 0.39) | 0.29 |
| Model 2 | 795 | −0.02 (−0.28, 0.25) | 0.89 | 0.11 (−0.17, 0.39) | 0.44 | 0.10 (−0.15, 0.36) | 0.43 |
| AC at 36 months |  |  |  |  |  |  |  |
| Model 1 | 831 | 0.06 (−0.23, 0.35) | 0.69 | 0.05 (−0.24, 0.33) | 0.74 | 0.06 (−0.23, 0.35) | 0.69 |
| Model 2 | 831 | 0.05 (−0.26, 0.36) | 0.76 | 0.17 (−0.17, 0.50) | 0.33 | 0.01 (−0.28, 0.30) | 0.94 |
| AC at 48 months |  |  |  |  |  |  |  |
| Model 1 | 758 | −0.14 (−0.51, 0.22) | 0.45 | −0.04 (−0.39, 0.30) | 0.81 | −0.003 (−0.38, 0.38) | 0.99 |
| Model 2 | 758 | −0.08 (−0.48, 0.32) | 0.68 | 0.001 (−0.40, 0.41) | 1.00 | −0.04 (−0.41, 0.34) | 0.84 |
| AC at 54 months |  |  |  |  |  |  |  |
| Model 1 | 758 | −0.09 (−0.47, 0.28) | 0.63 | −0.34 (−0.70, 0.02) | 0.07 | 0.19 (−0.20, 0.59) | 0.33 |
| Model 2 | 758 | −0.02 (−0.43, 0.39) | 0.94 | 0.03 (−0.39, 0.45) | 0.88 | 0.15 (−0.24, 0.53) | 0.46 |

^1^ All such values are *β* (95% CI) per 1 SD increment of maternal dietary pattern score. AC, abdominal circumference. Model 1 adjusted for exact age at each measurement. Model 2 further adjusted for infant sex, birth order, gestational age, duration of any breastfeeding, ethnicity, maternal age, height, pre-pregnancy BMI, weight gain until 26–28 weeks gestation, education level, gestational diabetes, energy intake, and scores of the other two dietary patterns (e.g., adjusting for SfN and PCB pattern scores for associations between VFR pattern and childhood adiposity).
